# Dynamics of Gut Microbial Community and Function in Growing Male Raccoon Dogs

**DOI:** 10.3390/ani16203238

**Published:** 2026-10-15

**Authors:** Shaochen Yu, Weixiao Nan, Chao Xu, Lei Sun, Chongshan Yuan, Zhipeng Li

**Affiliations:** 1 College of Animal Science and Technology, Jilin Agricultural University, Changchun 130118, China; 2 Key Laboratory of Animal Production, Product Quality and Security, Ministry of Education, Jilin Agricultural University, Changchun 130118, China

**Keywords:** raccoon dog, gut microbiota, growth period, metabolic function

## Abstract

**Background:** Raccoon dogs have short breeding cycles and strong adaptability, and their fur is economically valuable. Although the gut microbiota, a key regulator of animal health, has been extensively studied in livestock, its composition and function in raccoon dogs remain poorly understood. **Methods:** In this study, the gut microbiota structure and functional profiles of ten raccoon dogs were analyzed during their growth period, including 2 (M2), 3 (M3), 4 (M4), 6 (M6), and 8 (M8) months after birth. **Results:** Our findings revealed that the richness and diversity of the gut microbiota in growing raccoon dogs increased initially and then decreased with age. Significant intergroup separation was observed in principal coordinates analysis (PCoA) and non-metric multidimensional scaling (NMDS) analyses, while the unweighted pair–group method with arithmetic means (UPGMA) analysis indicated temporal fluctuations in microbial composition. At the phylum level, the dominant taxa were Firmicutes, Bacteroidota, Actinobacteriota, and Proteobacteria. At the genus level, the M2 group was enriched with *Ligilactobacillus* and *Megasphaera;* the M3 group with *Blautia*, *Megamonas*, *Peptoclostridium*, and *Prevotella 9*; the M4 group with *Cetobacterium* and *Streptococcus*; the M6 group with *Lactobacillus* and *Prevotella 9*; and the M8 group with *Prevotella 9* and *Turicibacter*. Function prediction of gut microbiota identified metabolism (particularly carbohydrate, amino acid, energy, and nucleotide metabolism) and genetic information processing (including replication and repair) as the predominant functional categories. **Conclusions:** The present results offer an understanding of how the gut microbiota of growing raccoon dogs changes over time.

## Simple Summary

As economically important fur-bearing animals, raccoon dogs are widely farmed around the world. Nevertheless, developing standardized breeding protocols to improve the survival of growing raccoon dogs remains a major challenge for the industry. The gut microbiota is essential for the health of young animals, and the early postnatal stage is a critical period for microbial colonization. However, research on the composition and function of the gut microbiota in raccoon dogs remains limited. In this study, we systematically collected fecal samples from raccoon dogs at five key developmental time points (2, 3, 4, 6, and 8 months of age) and characterized their gut microbial composition using 16S rRNA sequencing. The present findings provide theoretical support for the optimization of raccoon dog breeding management.

## 1. Introduction

Raccoon dogs (*Nyctereutes procyonides*) are widely distributed in East Asia and the Russian Far East and are known for their valuable pelage [1]. The dense fur and distinctive coloration of raccoon dogs make them particularly suitable for the production of premium fur products, including coats, hats, and other luxury apparel [2]. Recent decades have seen growing commercial demand for raccoon dog fur, driven by global fashion trends and the expansion of luxury markets [3]. Nevertheless, captive breeding remains difficult due to the carnivorous diet, aggressive temperament, and high stress susceptibility of this species. These behavioral characteristics necessitate the development of standardized husbandry protocols to ensure animal welfare and production efficiency. The growth phase represents a critical developmental window for juvenile raccoon dogs, during which proper nutrition and management directly affect fur quality and yield [4]. Maintaining the health of raccoon dogs during this period is therefore essential for commercial fur production.

The gut microbiota includes bacteria, archaea, fungi, viruses, phages, and eukaryotic parasites [5]. This microbial ecosystem exerts profound influences on host physiology by metabolizing nutrients [6], sustaining immune homeostasis [7], and modulating growth [8]. Balanced gut microbial composition and stable ecosystem dynamics are essential for proper digestive function, metabolic regulation, and reproductive health in animals. The protective roles of commensal microbiota are mediated through multiple pathways: stimulation of host immune responses, production of bioactive metabolites, and competitive exclusion of pathogenic microorganisms through colonization resistance [9]. As the host’s “second genome,” the gut microbiota exhibits dynamic plasticity in response to factors such as diet, physiological stress, host genetics, and health status [10,11]. Imbalances in gut microbial communities have been associated with various pathological states, from autoimmune disorders [12] and metabolic dysregulation [10,13] to increased susceptibility to infectious diseases [14]. These findings underscore the importance of maintaining microbial homeostasis for optimal raccoon dog husbandry. Notably, the gut microbiota undergoes significant compositional and functional transformations throughout host development [15,16]. It has been reported that there is a correlation between the quality of raccoon dog fur and gut microbiota [17]. Although previous studies have investigated the gut microbial ecology of raccoon dogs, they have mainly focused on specific growth stages. It remains unclear whether these findings can fully reflect the dynamic changes in the gut microbiota across the entire growth period. This knowledge gap hinders the development of evidence-based feeding strategies for this commercially important species.

In this study, fecal samples were collected from raccoon dogs at five key developmental time points (2, 3, 4, 6, and 8 months of age), and gut microbial composition was characterized by 16S rRNA sequencing, aiming to identify the core microbial communities across different growth stages and elucidate their predominant metabolic functions.

## 2. Materials and Methods

### 2.1. Sample Collection

The experiment was conducted at the Zuojia Fox and Raccoon dog Breeding Base in the Jilin Province. The breeding farm lies on gently sloped land and includes sufficient sunlight and good air circulation. The housing units adopt steel sheet roofs; the eaves are 1.5–2.0 m high, and each single unit covers a width of 2–4 m. The cages are reinforced with steel frameworks and enclosed by wire mesh. All raccoon dogs were raised individually in 1.0 m × 1.0 m cages, and no artificial temperature and humidity control system was equipped in the breeding facility. Feed was provided at 07:00 and 16:00 every day. The composition of the basal diet is presented in Table 1. A total of 10 healthy male raccoon dogs (4.12 ± 0.47 kg) with consistent growth rates and no history of disease were selected. The same 10 individuals were sampled repeatedly at 2 (M2 group, n = 8), 3 (M3 group, n = 8), 4 (M4 group, n = 8), 6 (M6 group, n = 8), and 8 (M8 group, n = 10) months after birth to track the periodic changes in their gut microbiota. At each sampling time point, 1–2 g of fresh feces was collected in the morning, approximately 2–3 h after the morning feeding. The collected fecal samples were placed in sterile centrifuge tubes and stored in a liquid nitrogen tank immediately after collection. After all samples at each stage were collected, they were transferred to the laboratory’s −80 °C freezer for long-term storage. After all the samples had been collected, they were processed and sequenced in a single batch.

### 2.2. DNA Extraction and PCR Amplification

Total DNA was extracted from fecal samples with the DNeasy PowerSoil kit (QIAGEN, Hilden, Germany). Subsequently, in order to obtain high-quality total DNA, the samples were centrifuged to remove impurities. Primers specific to conserved regions of the 16S rRNA gene were used for PCR amplification, and the products were purified with magnetic beads to remove small molecular byproducts. PCR product purity was assessed by 1% agarose gel electrophoresis. The V3/V4 regions were then amplified by PCR, verified on a 2% agarose gel [18], and prepared for sequencing by adding platform-specific adapters and sample-specific barcodes. The libraries were then pooled and sequenced on the Illumina platform.

### 2.3. 16S rRNA Sequencing Data Preprocessing

Raw tags were processed with Fastp (version 0.23.1) to obtain high-quality tag data. Cutadapt (version 1.9.1) was applied to remove primer sequences and reduce noise [19]. The sequences were then compared with the species annotation database (https://www.arb-silva.de/; 20 March 2025) to remove chimeric sequences, yielding the final valid data. ASVs with 97% sequence similarity were clustered together. Microorganisms were identified and classified using the QIIME2 software package version 1.8.0 platform [20,21]. The generated ASV table was exported to R-studio and subjected to ASV clustering, classification, and diversity assessment.

### 2.4. Alpha Diversity Analysis and Beta Diversity Analysis

QIIME2 software was used to estimate microbial richness and evenness. PCA, principal coordinates analysis (PCoA), non-metric multidimensional scaling (NMDS) analysis of similarities (ANOSIM), and permutational multivariate analysis of variance (PERMANOVA) were conducted to explore group differences [22]. The speccaccum function in the Vegan package of R-studio was used to generate species accumulation curves. UPGMA clustering maps were drawn using the UPGMA.tre function in QIIME2.

### 2.5. Significance Analysis of Intergroup Differences

The Kruskal–Wallis rank sum test was applied to screen species with differences in component abundance, and linear discriminant analysis (LDA) was used to calculate the effect size of each differential species. To investigate the potential interactions between gut microbiota in different age groups, Spearman correlation analysis was used to screen the top 25 species at the genus level for total abundance. Species with absolute correlation coefficients > 0.6 and significant *p*-values ≤ 0.05 were retained for further research.

### 2.6. Functional Prediction

PICRUSt (https://github.com/picrust/picrust2; 20 March 2025) was applied to predict the potential molecular functions of the microbial communities [23]. In order to analyze Tax4Fun, runRefBlast and make Functional Prediction functions were used, with a default reference database and a clustering threshold of 99% for analyzing 16S rRNA genes [24,25]. BugBase was used to predict bacterial composition in sequencing results [26]. Filtered sequences were used to classify sequences with the Silva 138 99% ASV full-length sequence database [27].

## 3. Results

### 3.1. The Richness and Diversity of Gut Microbiota

**Figure 1:**
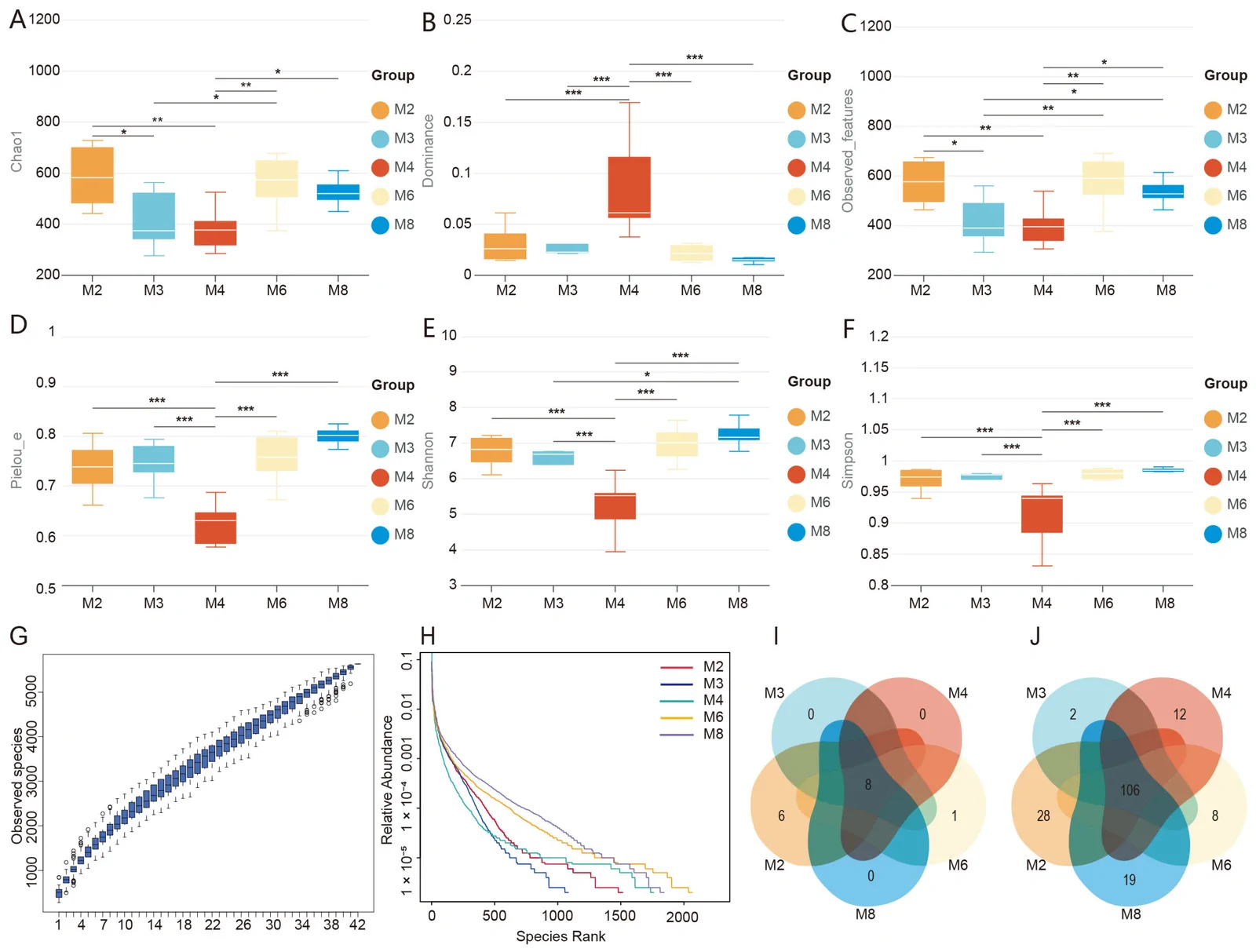
Gut microbiota richness and diversity (M2, 3, 4, 6: n = 8; M8: n = 10). (**A**–**F**): Alpha diversity indices among groups. (**G**): Species accumulation boxplot. (**H**): Rank abundance curve. (**I**): Venn diagram at the phylum level. (**J**): Venn diagram at the genus level. * *p* < 0.05; ** *p* < 0.01; *** *p* < 0.001.

As shown in Figure 1A,C, notable differences were observed in the diversity and richness of the gut microbiota among raccoon dogs of different ages, with the lowest Chao1 and Observed features in the M4 group, indicating that species abundance first decreases and then stabilizes with age. Similarly, the Shannon and Simpson were the lowest in the M4 group, indicating that community diversity first decreased and then increased with age (Figure 1E,F). The Pielou is used to assess species evenness (Figure 1D). Although the M4 group had the lowest Pielou value, the evenness of the gut microbiota increased significantly with age. In addition, the trend of the Dominance value is opposite that of the Pielou index, which further corroborates this result (Figure 1B). A species accumulation boxplot can describe the species composition and abundance of the sample. As the sample size increased, the boxplots tended to flatten, suggesting that the sample size was sufficient for data analysis (Figure 1G). Figure 1H shows that the curve became wider and flatter with age, suggesting that species abundance and evenness increased with age. Venn diagram analysis (Figure 1I,J) showed that the five groups shared eight microbial phyla. In addition, there were 6 microbial species unique to group M2, and 1 bacteria unique to group M6. At the genus level, the total number of species shared by the five groups was 106, with 28, 2, 12, 8, and 19 species unique to groups M2, M3, M4, M6, and M8, respectively.

### 3.2. Differences in Gut Microbiota

**Figure 2:**
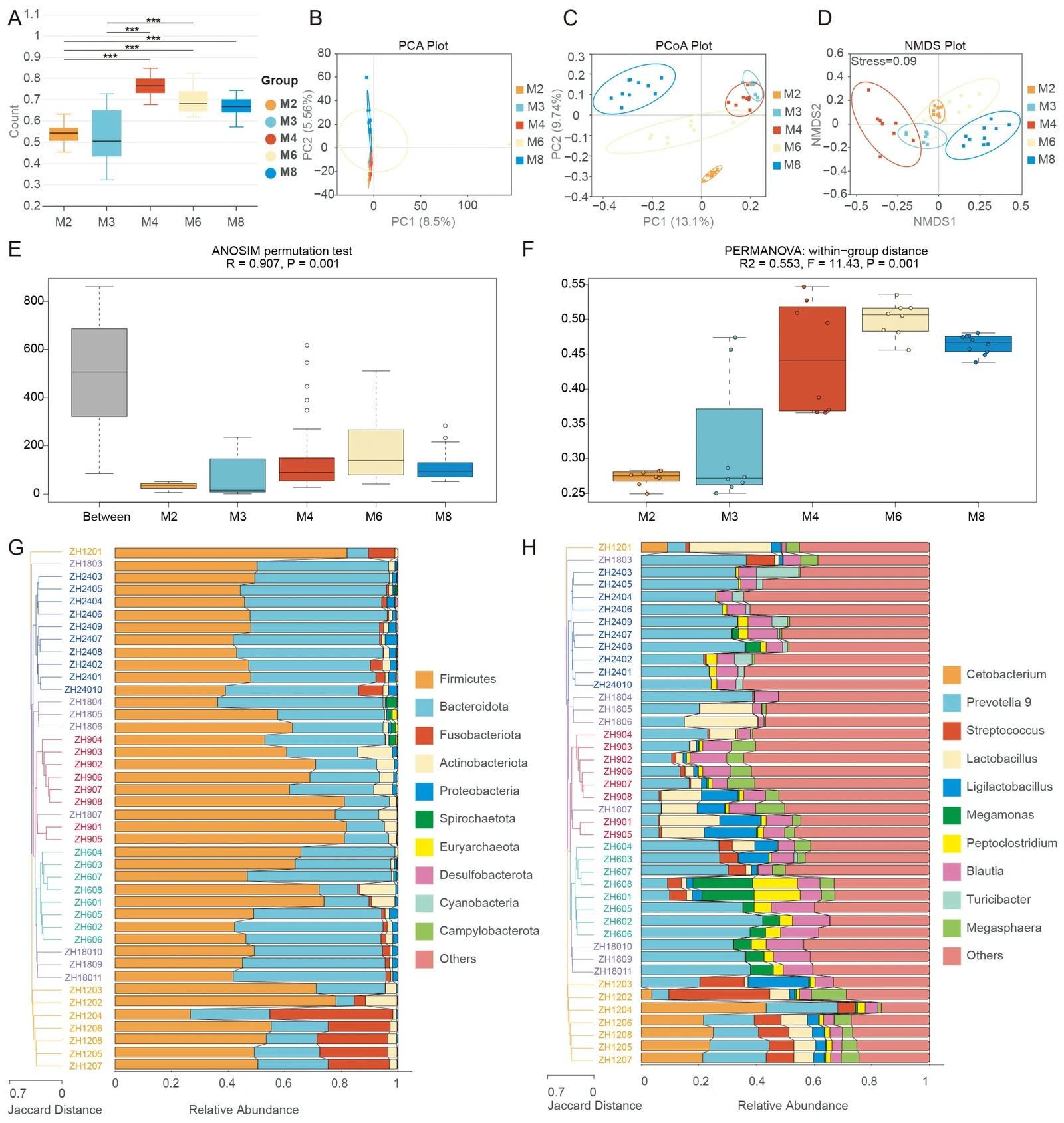
Differences in gut microbiota among different groups (M2, 3, 4, 6: n = 8; M8: n = 10). (**A**): Beta index intergroup difference test. (**B**): PCA analysis. (**C**): PCoA analysis. (**D**): NMDS analysis. (**E**): ANOSIM analysis. (**F**): PERMANOVA analysis. (**G**,**H**): UPGMA analysis at the phylum and genus level. M2: (ZH601-ZH608); M3: (ZH901-ZH908); M4: (ZH1201-ZH1208); M6: (ZH1803-ZH1807, ZH1809-ZH18011); M8: (ZH2401-ZH24010). *** *p* < 0.001.

As shown in Figure 2A, the M2 group differed significantly from the M4, M6, and M8 groups, while the M3 group differed significantly from the M4 and M6 groups. A beta index intergroup difference test is a statistical method used to compare differences between groups. It can be inferred that as age increased, the gut microbiota changed. Figure 2C,D shows clear group separation in the PCoA and NMDS plots. ANOSIM analysis confirmed significant divergence in gut microbial community structure across the groups (R = 0.907, *p* = 0.001), with greater dissimilarity between groups than within groups (Figure 2E). Figure 2F shows that PERMANOVA illustrated distinct differences in community homogeneity among groups (R2 = 0.553, F = 11.43, *p* = 0.001). ZH12 had the highest within-group variation, whereas ZH6 displayed the most homogenous gut microbial community. The similarity of gut microbiota and bacterial community structure between groups can be detected through the arithmetic mean unweighted pairwise method (UPGMA). The hierarchical clustering tree was shown in Figure 2G,H. The M2 (ZH601-ZH608) and M3 (ZH901-ZH908) groups clustered together, while the M4 (ZH1201-ZH1208) and M6 (ZH1803-ZH1807, ZH1809-ZH18011) groups were dispersed at both ends, and the M8 (ZH2401-ZH24010) group was closer to the M4 and M6 groups.

### 3.3. Gut Microbial Community Composition

**Figure 3:**
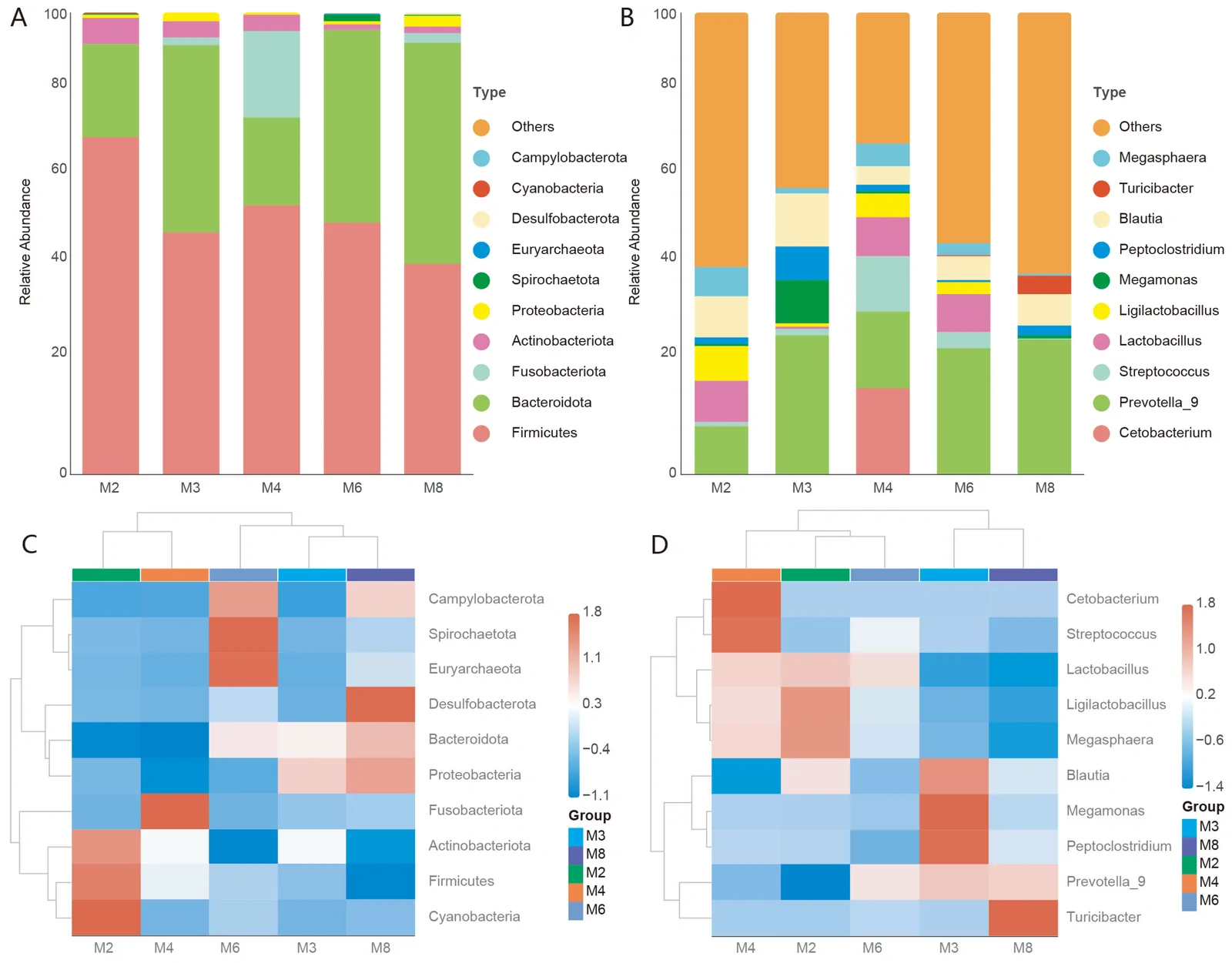
Gut microbiota community composition (M2, 3, 4, 6: n = 8; M8: n = 10). (**A**): Relative abundance at the phylum level. (**B**): Relative abundance at the genus level. (**C**,**D**): Clustered heatmaps of species abundance at the phylum and genus levels, respectively.

As shown in Figure 3A, Firmicutes, Bacteroidota, Fusobacteriota, Actinobacteriota, and Proteobacteria dominated the phyla across the groups. At the genus level, *Prevotella 9*, *Streptococcus*, *Lactobacillus*, *Peptoclostridium*, and *Blautia* were the dominant genera across the groups (Figure 3B). The species abundance clustering heatmap is used to discover the high or low aggregation content of species in each group. At the phylum level, Cyanobacteria, Firmicutes, and Actinobacteriota were enriched in M2; Fusobacteriota in M4; Euryarchaeota, Spirochaetota, and Campylobacterota in M6; Proteobacteria in M3; and Campylobacterota, Desulfobacterota, Bacteroidota, and Proteobacteria in M8 (Figure 3C). At the genus level, the species with higher abundance in the M4 group were *Cetobacterium* and *Streptococcus*; the species with higher abundance in the M2 group were *Ligilactobacillus* and *Megasphaera*; the species with higher abundance in the M6 group were *Lactobacillus* and *Prevotella 9*; the species with higher abundance in the M3 group were *Blautia*, *Megamonas*, and *Peptoclostridium*; and the species with higher abundance in the M8 group were *Prevotella 9* and *Turicibacter* (Figure 3D).

### 3.4. Differential Abundance of Gut Microbiota

**Figure 4:**
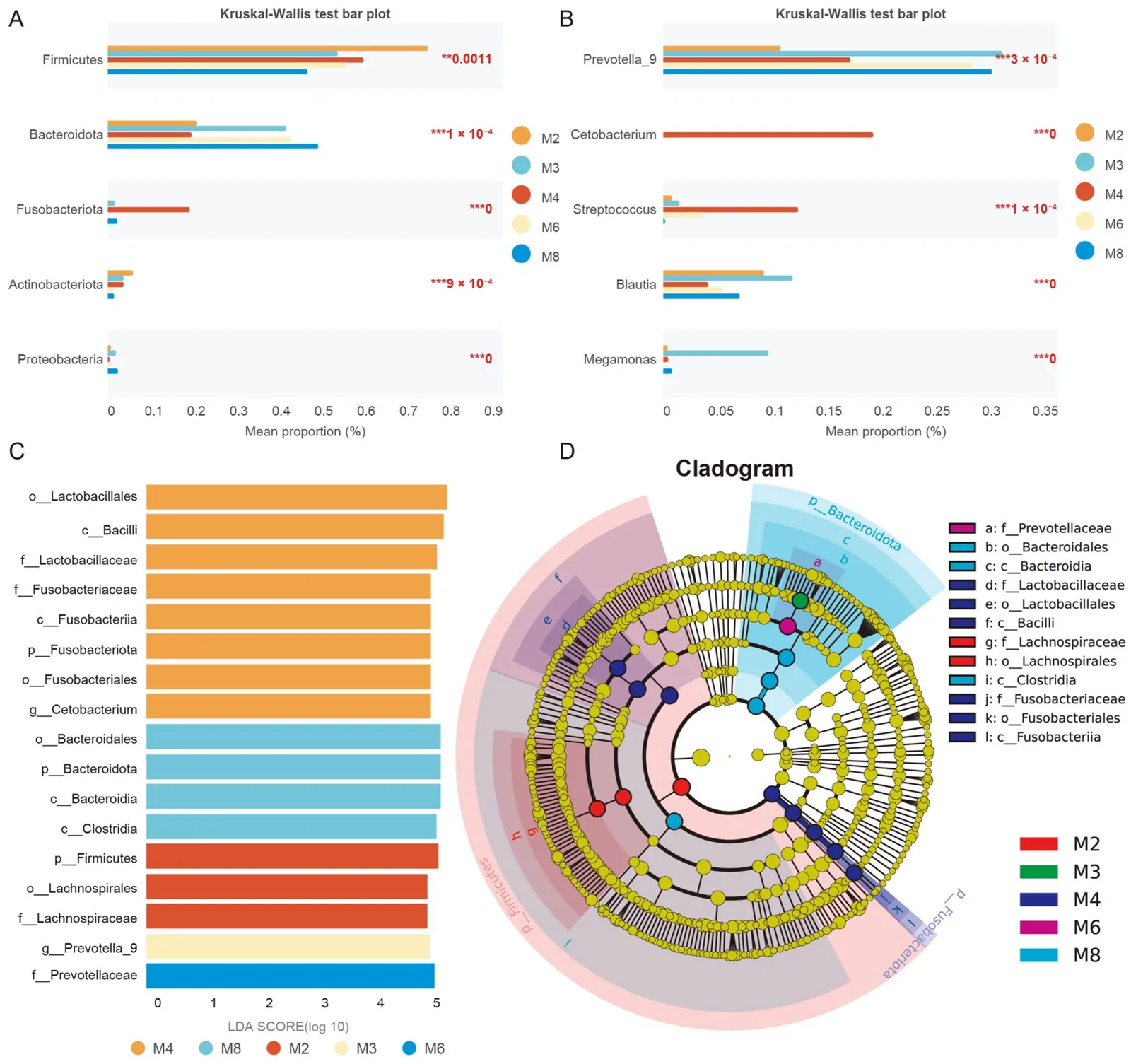
Differential abundance of gut microbiota among different groups (M2, 3, 4, 6: n = 8; M8: n = 10). (**A**): Kruskal–Wallis H-test bar plot at the phylum level. (**B**): Kruskal–Wallis H-test bar plot at the genus level. (**C**): LDA Score of Different Species. (**D**): Cladogram of Different Species. ** *p* < 0.01; *** *p* < 0.001.

As demonstrated by the Kruskal–Wallis H-test bar plots (Figure 4A,B), the differentially abundant phyla were Firmicutes, Bacteroidota, Fusobacteriota, Actinobacteriota, and Proteobacteria (Figure 4A), and the differentially abundant genera were *Prevotella 9*, *Cetobacterium*, *Streptococcus*, *Blautia*, and *Megasphaera* (Figure 4B). LEfSe analysis (LDA score > 4, *p* < 0.05) is shown in Figure 4C: the most enriched taxa in the M2 group were Firmicutes, Lachnospirales, and Lachnospiraceae, while *Prevotella 9* was enriched in the M3 group. The Lactobacillates Bacilli, Lactobacillaceae, Fusobacteriaceae, Fusobacteriia, Fusobacteriota, Fusobacteriales, and *Cetobacterium* were enriched in the M4 group. Prevotellaceae was the most enriched taxon in the M6 group, whereas the enriched taxa in the M8 group were Bacteroidales, Bacteroidota, Bacteroidia, and Clostridia. Figure 4D shows the evolutionary branching of different species. The radiating circles represent taxonomic levels from phylum to genus, and each small circle indicates a taxon at that level, with its diameter proportional to relative abundance. Taxa without significant differences are uniformly yellow, whereas those with significant differences are colored according to group.

### 3.5. Co-Occurrence Network Analysis of Microbial Interactions

**Figure 5:**
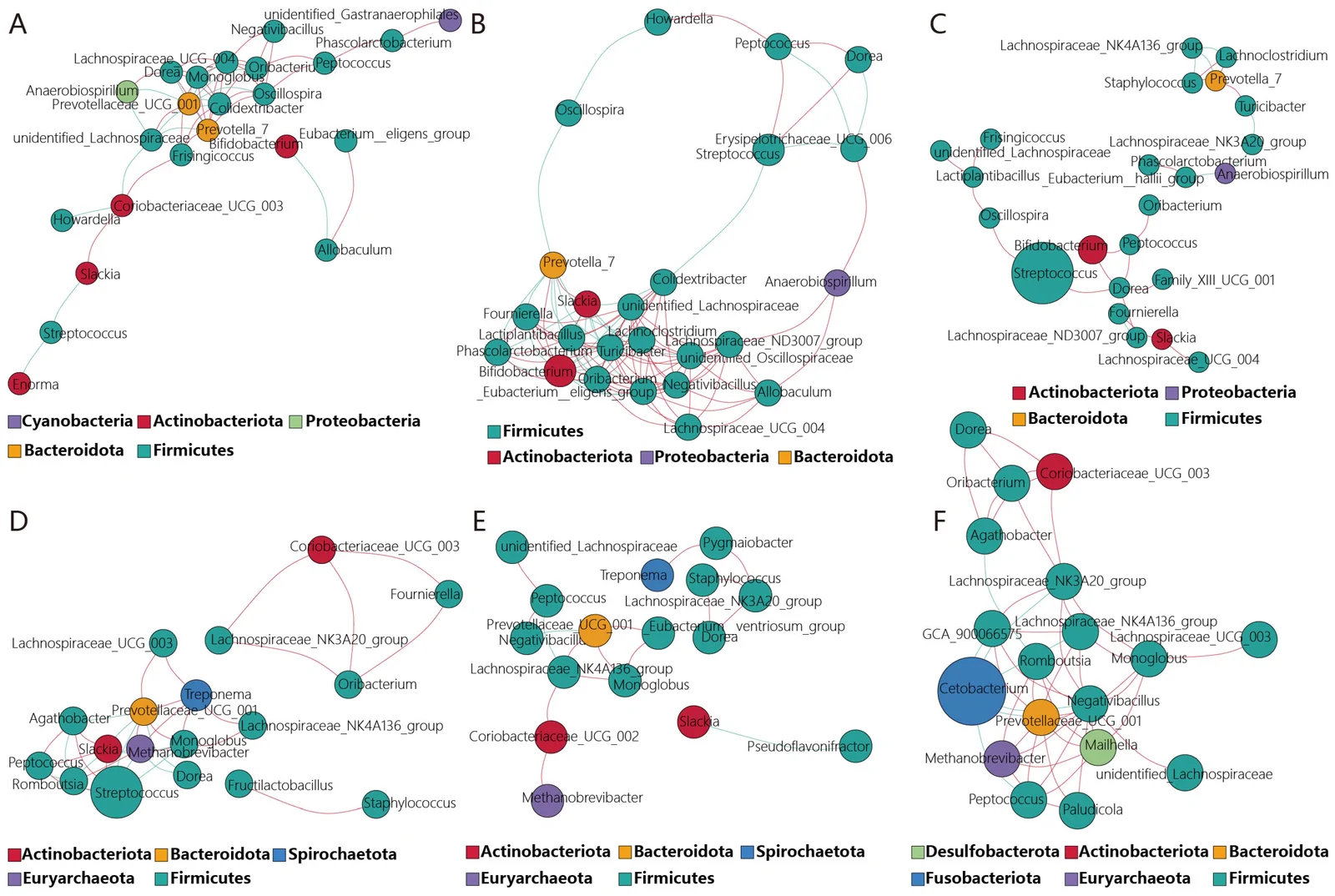
The co-occurrence network of the microbial community at the genus level (M2, 3, 4, 6: n = 8; M8: n = 10). (**A**): M2 group; (**B**): M3 group; (**C**): M4 group; (**D**): M6 group; (**E**): M8 group; (**F**): all groups. The red and green lines indicate positive and negative correlations, respectively.

The co-occurrence network showed that 73 edges including 57 positive edges and 16 negative edges were in the M2 group, while *Prevotellaceae UCG 001*, *Prevotella 7*, and *Monoglobus* contained numerous correlations (Figure 5A). Group M3 has 157 edges, including 116 positive edges and 41 negative edges, while *Lactiplantibacillus*, *Bifidobacterium*, *Turicibacter*, *Slackia*, and *Lachnoclostridium* contained numerous correlations (Figure 5B). Group M4 has 50 edges, including 43 positive edges and 7 negative edges, while *Dorea*, *Staphylococcus*, and *Prevotella 7* contained numerous correlations (Figure 5C). Group M6 has 62 edges, including 49 positive edges and 13 negative edges, while *Slackia*, *Streptococcus*, *Methanobrevibacter*, and *Romboutsia* contained numerous correlations (Figure 5D). Group M8 has 42 edges, including 39 positive edges and 3 negative edges, while the *Prevotellaceae UCG 001* and *Lachnospiraceae NK3A20 groups* contained numerous correlations (Figure 5E). There are 117 edges, including 103 positive edges and 14 negative edges in all groups, while *Prevotellaceae UCG 001*, *Negativibacillus*, *Cetobacterium*, *Mailhella*, and *Peptococcus* contained numerous correlations (Figure 5F).

### 3.6. Function Prediction of Gut Microbiota

**Figure 6:**
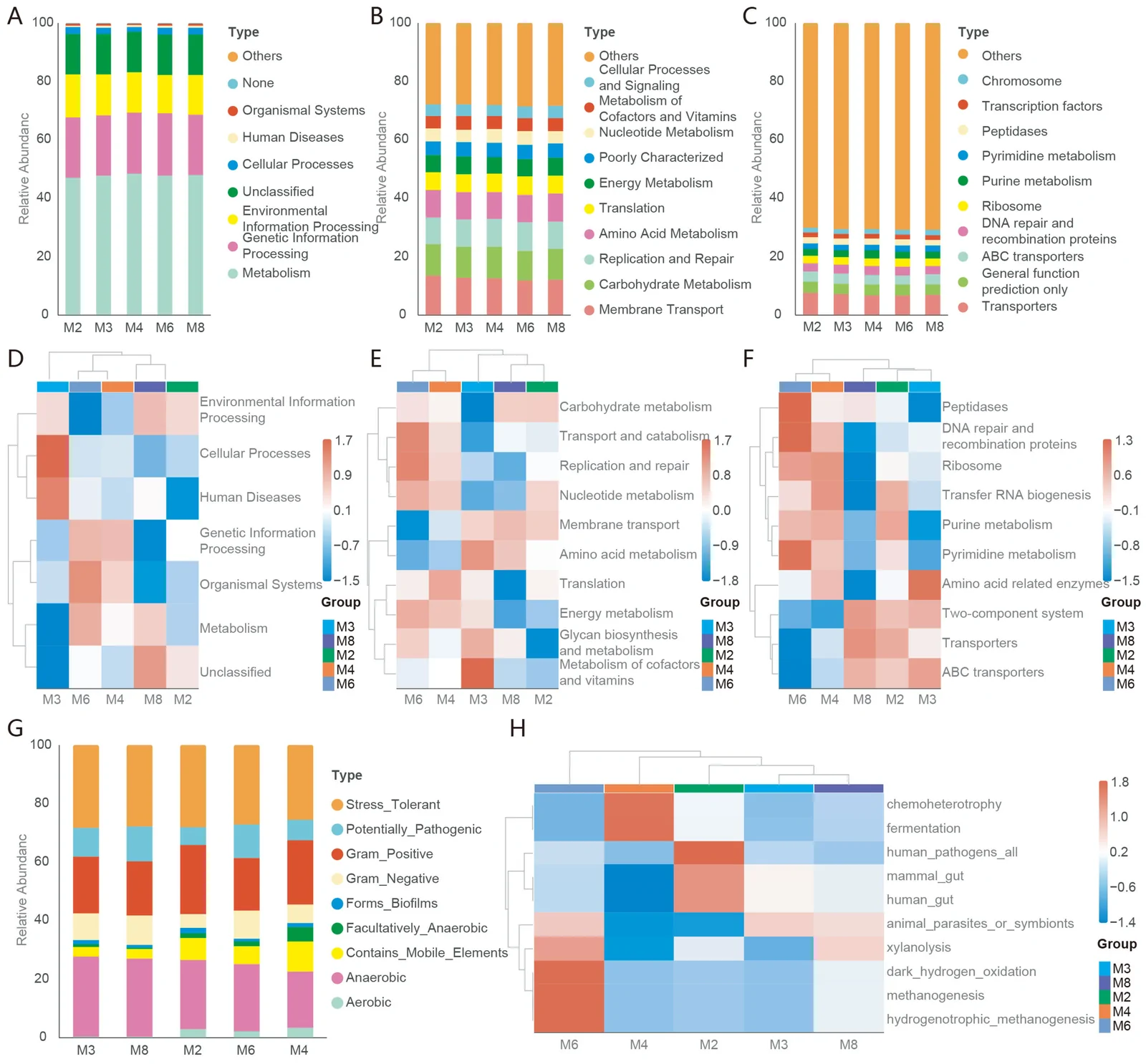
Function prediction of gut microbiota (M2, 3, 4, 6: n = 8; M8: n = 10). (**A**–**C**): PICRUSt analysis in different levels. (**D**–**F**): Tax4Funanalysis in different levels. (**G**): BugBase analysis. (**H**): FAPROTAX analysis.

The top 10 most abundant functions of the gut microbiota at different annotation levels were statistically analyzed using PICRUSt (Figure 6A–C). The dominant functions in level 1 were metabolism, genetic information processing, environmental information processing, cellular processes, human diseases, and organismal systems (Figure 6A). The main functions in level 2 were membrane transport, carbohydrate metabolism, replication and repair, amino acid metabolism, translation, energy metabolism, poorly characterized, nucleotide metabolism, metabolism of cofactors and vitamins, and cellular processes and signaling (Figure 6B). The main functions in level 3 were transporters, general function prediction only, ABC transporters, DNA repair and recombination proteins, ribosome, purine metabolism, pyrimidine metabolism, peptidases, transcription factors, and chromosome (Figure 6C). In addition, we obtained similar results by analyzing the function of gut microbiota through Tax4Fun. The functional differences among the groups have been demonstrated using heatmaps (Figure 6D–F). BugBase is a tool based on 16S rRNA for predicting microbial phenotype and performing intergroup differential analysis. It can predict seven aspects of information, including Gram positive/negative, biofilm formation, pathogenic potential, aerobic/anaerobic, stress tolerant, facultatively anaerobic, and contains mobile elements. Through BugBase analysis, our results indicated that the functions of each group were similar (Figure 6G). Function Annotation of Prokaryotic Taxa (FAPROTAX) is a database developed by the Louca Laboratory of the Department of Biology at the University of Oregon in the United States. The database utilizes current literature on cultured strains to predict functional annotations of prokaryotes such as genera or species. Our results showed that the core functions of each microbiota were mainly chemoheterotrophy, fermentation, animal parasites or symbionts, mammal gut, and human gut (Figure 6H).

## 4. Discussion

At present, the dynamic changes in the gut microbiota of raccoon dogs during their growth period are still unclear. This study systematically investigates the compositional dynamics and functional potential of gut microbiota across growth stages, with the ultimate goal of establishing an evidence-based framework for improved husbandry practices in commercial raccoon dog breeding operations. Alpha diversity metrics, including species richness, diversity, and dominance, collectively characterize the microbial complexity within gut ecosystems [28]. Our analysis revealed a distinct temporal pattern in the gut microbiota of raccoon dogs: diversity and richness initially decreased, then increased, and ultimately stabilized during the growth period. This developmental trajectory is consistent with the age-dependent microbial succession reported in other mammalian species [29]. Beta diversity analysis quantified compositional differences between developmental stages [30]. Significant microbial community shifts were detected between early and late growth phases, with PCoA, NMDS, ANOSIM, and PERMANOVA demonstrating clear temporal separation [31]. Hierarchical clustering analysis reinforced these findings, revealing time-dependent restructuring of microbial communities that parallels documented patterns of gut microbiota maturation [32].

Our results indicate that the gut microbiota was predominantly composed of Firmicutes, Bacteroidota, Actinobacteriota, and Fusobacteriota, consistent with previous findings in raccoon dogs [17]. It is worth noting that the gut microbiota mainly consists of Firmicutes and Bacteroidetes, a characteristic distribution pattern observed across mammalian gut ecosystems [33]. The Firmicutes/Bacteroidetes (F/B) ratio is positively correlated with energy absorption [34,35,36]. In this study, the F/B ratio was relatively high during the early growth stage, which may be related to the rapid absorption of energy from the diet to meet the high energy demand for growth. As growth slowed down with age, the F/B ratio gradually decreased. When the raccoon dogs stopped growing at the 8th month, the energy demand further declined, which may also contribute to the decrease in the F/B ratio. Although Actinobacteria have relatively low abundance in the gut microbiota, their decreased abundance in maintaining microbial balance, host immune regulation, and metabolic health can serve as a microbial marker for various chronic diseases [37]. A striking temporal pattern emerged for Fusobacteriota, with a significant increase in abundance at 4 months, which may be beneficial for the synthesis of short chain fatty acids, thereby maintaining the energy supply of intestinal epithelial cells and maintaining barrier integrity [38]. Genus-level analysis identified *Prevotella 9* as the predominant taxon across all age groups. As a beneficial member of the Bacteroidetes phylum, *Prevotella* can efficiently ferment complex polysaccharides such as resistant starch and pectin [39]. Furthermore, short-chain fatty acids (SCFAs) derived from Prevotella improve intestinal barrier function by increasing tight junction protein expression [40]. Notably, we observed a transient bloom of *Cetobacterium* abundance in the fourth month. Studies have found that *Cetobacterium* can significantly increase the activity of trypsin, lipase, and brush border enzymes, and supply energy to the intestinal epithelium by producing short-chain fatty acids. In addition, *Cetobacterium* can upregulate the expression of ZO-1 and Occludin, maintaining intestinal health [41]. Co-occurrence network analysis further revealed that *Cetobacterium* is correlated with multiple microorganisms, possibly because it enhances its interactions with the gut microbiota by producing vitamin B12 [42]. However, by 6 months of age, *Cetobacterium* abundance declined significantly, coinciding with the re-establishment of *Prevotella 9* dominance.

Although the host–modulatory effects of the gut microbiota are well established, the underlying mechanisms remain incompletely understood [43]. Moreover, while 16S rRNA sequencing effectively profiles microbial composition, it provides limited functional insights. To address this, functional prediction across developmental stages was performed using both Tax4Fun and PICRUSt. Notably, PICRUSt’s utility is constrained by its reliance on outdated reference databases [44], whereas Tax4Fun demonstrates superior predictive accuracy through its refined algorithm and updated genomic repositories [45]. Our dual-analysis approach consistently identified metabolism and genetic information processing as the predominant functional categories.

While this 16S rRNA study provides foundational insights into the dynamics of the raccoon dog gut microbiota, several limitations warrant consideration. During sequencing, biases arising from DNA extraction, PCR amplification, and primer selection, together with limited resolution at the species/strain level and relative abundance data that may not necessarily reflect absolute cell count or activity, constrain the accuracy of the results [46]. Exploring the periodic changes in the gut microbiota of growing raccoon dogs can help identify optimal feeding stages and provide a scientific basis for adjusting diet and management practices to improve health and growth performance. However, this study did not measure growth performance or fur quality; therefore, the relationship between these parameters and the gut microbiota of growing raccoon dogs remains to be explored in future research. Future investigations should also incorporate individual variation analysis, dietary habit correlations, and the integration of livestock management metadata. Such multidimensional approaches will enable a more robust understanding of microbiome–host interactions in commercial fur animal production.

## 5. Conclusions

Our results revealed significant stage-specific variations in the gut microbiota composition of growing raccoon dogs. At the phylum level, the dominant species of growth raccoon dogs at different times were Firmicutes, Bacteroidota, Actinobacteriota, and Proteobacteria. Genus-level analysis demonstrated significant differential abundance of several key taxa including *Prevotella 9*, *Megasphaera*, *Holdemanella*, *Romboutsia*, and *Fusionnibacter* across growth stages. Functional prediction analysis showed that these microbial communities were mainly involved in carbohydrate, amino acid, and energy metabolism.

## Figures and Tables

**Table 1:** Composition of basal diets (DM basis, g/kg).

Ingredients	Content
Extruded corn	405.00
Soybean meal	210.00
Meat meal	50.00
Fish meal	25.00
Blood meal	50.00
Distillers dried grains with solubles	100.00
Corn germ meal	100.00
Soybean oil	35.00
CaHPO_4_	8.00
Limestone powder	7.00
Premix	10.00

Metabolic energy and crude protein levels were 12.05 MJ/kg and 25.23%, respectively (calculated values). The crude protein levels of soybean meal, meat meal, and corn germ meal were 44.2%, 54.0%, and 17.8%, respectively.

## Data Availability

All data and materials are available from the corresponding authors upon request.
